# Assessing the effects of different agro-residue as substrates on growth cycle and yield of *Grifola frondosa* and statistical optimization of substrate components using simplex-lattice design

**DOI:** 10.1186/s13568-018-0565-8

**Published:** 2018-03-23

**Authors:** Bing Song, Jianqiang Ye, Frederick Leo Sossah, Changtian Li, Dan Li, Lingsi Meng, Shuai Xu, Yongping Fu, Yu Li

**Affiliations:** 1 Engineering Research Centre of Chinese Ministry of Education for Edible and Medicinal Fungi, Jilin Agricultural University, Changchun, 130118 People’s Republic of China; 2 Microbiology Research Institute, Guangxi Academy of Agricultural Sciences, Nanning, 530007 Guangxi People’s Republic of China

**Keywords:** *Grifola frondosa*, Crop straw, Simplex-lattice design, Yield and growth cycle, High-yielding formula, Biological efficiency

## Abstract

*Grifola frondosa* is an economically important edible and medicinal mushroom usually produced on substrate consisting of sawdust supplemented with wheat bran. Cultivation of *G. frondosa* on crop straw (corn cob, corn straw, rice straw, and soybean straw) as a substrate was optimized by using the D-optimum method of the simplex-lattice design, and the alternative of crop straw as a substitute for sawdust in the substrate composition was determined by the optimized model. The results showed that there was a significant positive correlation existing between the yield and corn cob. The growth cycle was negatively correlated with sawdust, corn cob and soybean straw, with sawdust significantly shortening the growth cycle of *G. frondosa*. The optimized high-yielding formula included 73.125% corn cob, 1.875% rice straw, 23% wheat bran and 2% light calcium carbonate (CaCO_3_) (C/N = 48.40). The average yield of the first flush was 134.72 ± 4.24 g/bag, which was increased by 39.97% compared with the control formula. The biological efficiency (BE) was 44.91 ± 1.41%, which was increased by 38.53% compared with the control. Based on the results of this study, corn cob can replace sawdust as one of the main cultivation substrates of *G. frondosa*.

## Introduction

*Grifola frondosa* (Dicks.) Gray, named “Maitake” in Japan, is a rare edible and medicinal fungi mainly cultivated in China, Japan and Korea (Park et al. 2015). It has a high nutritional composition with compounds such as polysaccharides, proteins, unsaturated fatty acids, vitamins, and trace elements (Gu et al. 2007; Illana-Esteban 2008; Montoya et al. 2012). It also has a wide range of bioactive compounds (Shin and Lee 2014) which has been reported to have hypoglycaemic effects, anti-cancer properties, antiviral properties, antioxidant effects, immunomodulatory effects, anti-inflammatory activities, anti-cholesterol or cholesterol-lowering effects, as well as the ability to reduce blood pressure and protect the liver (Vetvicka and Vetvickova 2014; Ding et al. 2016; Lin et al. 2016). Due to the unique properties of *G. frondosa,* it has become a regular healthy food for consumption with a high demand in many countries such as the USA, Canada, Australia and some countries in Europe (Mayell 2001). The commercial profitability of this mushroom has significantly increased, necessitating a rapid expansion in its cultivation in China and other parts of the world.

*Grifola frondosa* is a wood-rot fungus, which grows on standing and dead wood. It acquires nutrients for growth and development by the degradation of lignocellulosic materials (Montoya et al. 2012; Yang et al. 2013a). Conventional cultivation is on deciduous hardwood sawdust or a mixture of assorted sawdust and cottonseed hulls (Ainsworth et al. 2008). However, recent efforts concerning the protection of forest resources, together with the high demand and rising price of both sawdust and cottonseed hulls have posed a challenge to commercial cultivation of *G. frondosa* using sawdust.

It is therefore important to find the above substrate alternatives that are equally suitable and cost-effective for the commercial production of *G. frondosa*. Currently, large tonnages of unexploited lignocellulosic agro-residues are available globally. For example, in China agro-residues are abandoned to decay in the field and sometimes disposed of through mass burning which is a major cause of air pollution in China (Yang et al. 2012; Hu et al. 2017; Teng et al. 2017). Utilization of these by-products as substrates for cultivating mushrooms will offer one of the best solutions for converting these inedible organic wastes into important edible biomass. At present, corn straw, soybean straw, rice straw and corn cobs are widely used in the cultivation of edible mushrooms, including *Lentinula edodes*, *Pleurotus ostreatus* and *Pleurotus eryngii* (Moonmoon et al. 2010; Yang et al. 2013b; Hoa et al. 2015; Pedri et al. 2015). Few studies exist where straw or straw mixtures were used as the cultivation substrates of *G. frondosa*; also only coffee grounds (Barreto et al. 2008) and olive oil residues (Gregori et al. 2009) have been reported as substrates for *G. frondosa*. But these two substrates are subject to the restrictions of the place of production and thus have not made gains in promotion and application. Also, these substrates are extremely variable and traditional techniques often do not guarantee a standardized product.

Mushroom growers commonly add different ratios of natural nutrient sources from corn meal, wheat bran and rice bran to sawdust to enhance *G. frondosa* colonization and formation of superior quality fruiting body (Montoya et al. 2012). In order to produce consistent high yield and maintain/increase profitability, a commercial grower must determine the processing conditions. These conditions include quality spawn, temperature, humidity and consider the addition of an additive that is readily available, cheap and proven to increase yield and produce high quality *G. frondosa* in a short period of time (Montoya et al. 2012; Kraisit et al. 2017). Mixture design is one of the most widely used methods for screening formulas (the sum of the main ingredients is a unit, 1 or 100%) and has been widely used in the feed, medicine and food industries (Kraisit et al. 2017; Pires et al. 2017; Saoudi et al. 2017). Among all mixture-design methods, the simplex-lattice design is the most commonly used method for substrate screening; it allows for good analysis of correlations of the different factors and the target values, and with regression analysis, the quantitative relations between the different substrates and assessment indicators can be obtained (Yang et al. 2016). The optimized formula and the impact of each substrate on the indicators can then be determined. Although some published studies (Shen and Royse 2001) have dealt with the effect of cereal agro-residue on growth and yield of *G. frondosa*, not much data is available on the use of statistical modelling for optimization of substrate composition to standardize yield in commercial cultivation as well as the presentation of alternatives to sawdust for sustainable production of *G. frondosa.*

The aim of the present study was to evaluate different agro-residues (sawdust, corn cob, rice straw, soybean straw and corn straw) as a substrate for cultivation of *G. frondosa;* and use simplex-lattice design method to model, optimize substrate composition and evaluate the combined effects on growth and yield of *G. frondosa*. Also, the agro-residue that can replace sawdust in the model for *G. frondosa* cultivation was investigated, thereby providing a reference for the further development and utilization of agricultural wastes.

## Materials and methods

### Study area

The study was conducted in Jilin Agricultural University, Changchun, China. The mycelial culture on potato dextrose agar slants, pre-screening of mycelial growth, and extracellular lignocellulolytic enzymes activity profiles were conducted at the Engineering Research Centre of the Chinese Ministry of Education for Edible and Medicinal Fungi, Jilin Agricultural University, Changchun, China. Whereas substrate preparation, fruiting body cultivation and optimization of fruiting body culture development were completed in the Mushroom base of Jilin Agricultural University, Changchun, China.

### Fungal culture and spawn preparation

The pure culture of *G. frondosa* H21 (ACCC52310) used in this study was obtained from the Institute of Natural Resources and Regional Planning, Chinese Academy of Agricultural Sciences, Beijing, China. It is a mid-temperature tolerant and moderate-yielding strain. The strain H21 was selected because it is a stable commercial strain in Jilin Province, China and has been well studied for mycelial growth characteristics, fruiting body development, genetic distinctness and extracellular lignocellulolytic enzymes activity. Hence, the difference in growth and yield of this strain as a result of different substrates used can clearly be discriminated. The stock culture was maintained on a potato dextrose agar (PDA) slants and incubated for seven days at a temperature of 25 °C and cultured slants were stored at 4 °C, until further use. *G. frondosa* liquid spawn was prepared by culturing (0.5 cm^2^) mycelial plugs in 500 ml Erlenmeyer flasks containing 200 ml Yeast malt broth (YM liquid medium), and incubated at 25 °C with shaking (120 rpm) on a rotatory shaker for 20 days. The YM liquid medium consisted of yeast extract 2.0 g/l, malt extract 20.0 g/l, corn flour 25.0 g/l, peptone 5.0 g/l, KH_2_PO_4_ 5.0 g/l, MgSO_4_ 2.5 g/l, sucrose 25.0 g/l, vitamin B_1_ 25 mg/l and nature pH.

### Substrates preparation

The various agro-residues (oak wood sawdust, corn cobs, corn straw, soybean straw and rice straw) and supplements [wheat bran and light calcium carbonate (CaCO_3_, average particle size is 1–3 μm)] for the cultivation of *G*. *frondosa* were purchased from the Mushroom base of the Jilin Agricultural University, Changchun, China. All agro-residues were chopped into pieces, dried, and ground into fine powder.

### Substrate formulation

The various independent variables were first studied using one-factor-at-a-time method to determine the initial range of the variables. This was achieved by preparing the substrates from the various agro-residues components first in petri dishes consisting of single substrate component with sterilized water added to ± 62.5% prior to autoclaving at 121 °C for 1 h, allowed to cool and inoculated with 5 cm^2^ mycelial plug to determine the radial mycelial growth rate followed by fruiting body cultivation in bags. Based on the preliminary results the variables (*X*_*1*_ = sawdust, *X*_*2*_ = corn cobs, *X*_*3*_ = soybean straw, *X*_*4*_ = rice straw and *X*_*5*_ = corn straw) were selected for the simplex-lattice design. These were then mixed in various combinations to determine a good fruiting substrate for the cultivation of *G. frondosa*.

A total of 21 mixture schemes were designed, and the design details are shown in Table 1. All possible combinations (mixtures) of the proportions from this equation were used. The 21 formulae were prepared following the 21 mixture schemes listed in Table 1; with the main ingredient accounting for 75%, scheme 1 is the control substrate mixture (CK) composed of 75% hardwood sawdust, 23% wheat bran and 2% light CaCO_3_, with water content of materials adjusted to 62.5%, with pH 7. The substituting ingredients were oak wood sawdust, corn cobs, corn straw, soybean straw and rice straw. All formulae contained 23% wheat bran, 2% light CaCO_3_ and 62.5% water with pH 7.0 and environmental conditions remained constant for all. All experiments were tested in three replicates and randomized to minimize the effects of unexpected effects in the observed response.

**Table 1:** Formula design of the cultivation materials and C/N

Formulas	Substrate mixture ratio					C/N
	X_1_ (%)^a^	X_2_ (%)^b^	X_3_ (%)^c^	X_4_ (%)^d^	X_5_ (%)^e^	
1 (CK)	100	0	0	0	0	74.64
2	0	100	0	0	0	48.9
3	0	0	100	0	0	20.51
4	0	0	0	100	0	34.88
5	0	0	0	0	100	32.25
6	50	50	0	0	0	59.12
7	50	0	50	0	0	31.83
8	50	0	0	50	0	47.22
9	50	0	0	0	50	44.24
10	0	50	50	0	0	28.65
11	0	50	0	50	0	40.58
12	0	50	0	0	50	38.49
13	0	0	50	50	0	26.71
14	0	0	50	0	50	25.22
15	0	0	0	50	50	33.48
16	60	10	10	10	10	47.66
17	10	60	10	10	10	40.6
18	10	10	60	10	10	25.82
19	10	10	10	60	10	34.88
20	10	10	10	10	60	33.48
21	20	20	20	20	20	14.9

^a^X_1_—sawdust

^b^X_2_—corn cob

^c^X_3_—soybean straw

^d^X_4_—rice straw

^e^X_5_—corn straw

### Model analysis

The Scheffé model was fitted using a polynomial quadratic equation in order to correlate the response variable (*Y*) to the independent variables (*X*). The Eq. (1) is as follows:

1 $$ Y = \mathop \sum \limits_{1 \le i \le q} \beta_{i} X_{i} + \mathop \sum \limits_{1 \le i < j \le q} \beta_{ij} X_{i} X_{j} $$

In a mixture experiment, it is not the volume of the actual amount of the single ingredient that matters, but rather its proportion in relation to other ingredients. The sum of all the ingredients is a constant total *T*, which is equal to 100% or 1 unless any constant mixture factors are present (Lundstedt et al. 1998).

Therefore, if *X*_1_, *X*_2_, *X*_3_, …*X*_*i*_ denote the proportions of components of a mixture, then:

2 $$ {\text{S}}^{q - 1}:\mathop \sum \limits_{{{\text{i}} = 1}}^{\text{q}} {\text{X}}_{\text{i}} = 1,0 \le {\text{X}}_{\text{i}} \le 1,\;{\text{i}} = 1,2, \ldots ,{\text{q}} $$

and

3 $$ X_{1} + X_{2} + X_{3} + X_{4} + X_{5} = 100\% \,\left( {i.e.,\,1} \right) $$

where *X*_1_ represents sawdust, *X*_2_ represents corn cob, *X*_3_ represents soybean straw, *X*_4_ represents rice straw, and *X*_5_ represents corn straw.

### Optimization design

Simplex lattice (D-Optional method) design was employed to determine the optimum mixture ratio, the number of experimental runs and the proportion of five agro-residues in each experimental run (Table 1) using the Design-Expert 8.05b software (Scheffé 1963; Yang et al. 2016).

### Substrate analyses

The carbon (C) and nitrogen (N) contents of the various substrate mixtures were measured using the furnace (Wang et al. 2015) and the Kjeldahl method (Verma et al. 2007) respectively. The carbon to nitrogen ratio of each substrate was calculated and are shown in Table 1.

### Cultivation method

For each formula, the ingredients, the wheat bran and the light CaCO_3_ were first mixed evenly per the respective mixture ratio scheme; water was then added to adjust the moisture content to approximately 62.5%. A total of 800.0 g of such mixed substrates (dry material 300.0 g) was used to fill a 33 cm × 17.5 cm × 0.05 cm polypropylene bag, with a 2.5 cm collar at the bag mouth. A column-shaped Polyvinylchloride (PVC), 2.5 cm in diameter and 12 cm in depth, was inserted into the collar to allow even distribution and colonization of the mycelia. The bags were heat sterilized by autoclaving at a temperature of 121 °C for 85 min and then cooled to room temperature. Further, each cooled bag was inoculated with 10 ml liquid spawn of *G. frondosa*. Upon inoculation, incubation occurred at 23–26 °C, the carbon dioxide (CO_2_) concentration at < 3000 ppm and in darkness, until the formation of primordia. Next, the breathable cover (diameter: 38 mm) was removed, and the bag was transferred to a fruiting chamber where temperature was maintained at 19–21 °C, the humidity at 93–97%, the carbon dioxide (CO_2_) concentration at 400–700 ppm and the light at 200 l×/12 h. After fruiting harvest, the yield of each bag was recorded (*only the first flush*); 10 bags from each mixture scheme were used for statistical analysis.

### Data collection

Data collected include growth cycle (time of inoculation to the first harvest of the fruiting body), growth parameters (length and width of a single fruiting body using a Vernier caliper), Yield parameters including total fresh weight (g) of mushroom and number of fruiting bodies per bunch were also recorded at harvest time. The BE was calculated as follows:

$$ BE \left( \% \right) = \frac{Fresh\,weight\,of\,mushroom}{Dry\,weight\,of\,substrate} \times 100 $$

### Statistical analysis

The inoculated bags were arranged in complete randomized block design comprising of each treatment in the growth room. All experiments were in three replicates. Design Expert statistical software package version 8.05 b was used for regression analysis. The Scheffé quadratic polynomial regression model was used to perform a quadratic multiple regression fitting of the yield per bag and the growth cycle against the formula ingredients, and a quadratic regression model of the yield and growth cycle was constructed against each ingredient in the mixture scheme. The effects of each ingredient on the yield and on the growth cycle were analyzed, and the correlations between each ingredient and the yield and between each ingredient and the growth cycle were analyzed by SPSS Statistics 17. The optimization function was used to set the variation range and the expected response value of each ingredient. Beginning with a random combination, the steepest slope prediction was performed until the target response value was reached. The optimized yield formula derived from the regression analysis was verified, and ANOVA analysis was performed to estimate statistical parameters using SPSS (Yang et al. 2016).

## Results

### Effects of different straw formulations on yield

By statistical analysis of the yield for each formula (Table 2), the regression model equation between the yield and each ingredient was developed as follows:

$$ Y = 96.97X_{1} + 133.95X_{2} + 87.31X_{3} + 31.60X_{4} + 78.08X_{5} + 14.76X_{1} X_{2} + 16.90X_{1} X_{3} - 102.94X_{1} X_{4} - 2.17X_{1} X_{5} - 18.16X_{2} X_{3} + 110.01X_{2} X_{4} + 44.18X_{2} X_{5} + 29.74X_{3} X_{4} + 73.71X_{3} X_{5} - 217.52X_{4} X_{5} \left( {R^{2} = 0.9956} \right). $$

**Table 2:** Measured values and predicted values of yield

Formulas^1^	Yield (g)		Formulas	Yield (g)	
	Measured value^2^	Predicted value		Measured value	Predicted value
1 (CK)	97.25 ± 9.71^def^	96.97	12	116.75 ± 7.57^bc^	117.06
2	134.25 ± 14.59^a^	133.95	13	66.40 ± 10.56^h^	66.89
3	87.40 ± 12.36^fg^	87.31	14	100.6 ± 9.15^de^	101.12
4	31.75 ± 3.61^i^	31.60	15	0.00 ± 0.00^j^	0.46
5	78.20 ± 20.00^gh^	78.08	16	86.65 ± 10.00^fg^	87.09
6	119.00 ± 17.45^b^	119.15	17	116.00 ± 8.00^bc^	116.79
7	96.00 ± 8.75^ef^	96.37	18	93.50 ± 9.50^f^	91.04
8	38.25 ± 2.56^i^	38.55	19	50.50 ± 10.05^hi^	49.04
9	86.65 ± 12.98^fg^	86.99	20	78.20 ± 11.5^g^	76.23
10	105.75 ± 18.15^cde^	106.09	21	86.65 ± 12.5^fg^	83.52
11	110.00 ± 11.41^bcd^	110.27			

^1^Notes are the same as Table 1

^2^Means followed by the same letter are not significantly different at a level of 5% (P < 0.05)

With the regression model, the predicted yield can be calculated for each formula, and the measured yield and the predicted yield were basically the same. The statistical analysis showed that the P values of the mixed linear model and the quadratic regression model were both less than 0.0001, indicating that these two models were very significant (P < 0.0001) and well fitted the relationship between ingredients and yield. In addition, the correlation coefficient (R^2^ = 0.9956) indicated that the equation model had 99.56% goodness-of-fit with the experimental data (Tables 2 and 3).

**Table 3:** Variance analysis of the quadratic polynomial regression model for yield

Variation sources	Sum of squares	DF	Mean square	F	P
Model	3,760,676	14	2686.20	397.07	< 0.0001
Linear mixed model	29,142.60	4	7285.65	1076.96	< 0.0001
X_1_X_2_^a^	14.08	1	14.08	2.08	0.0079
X_1_X_3_	18.01	1	18.01	2.66	0.0027
X_1_X_4_	918.75	1	918.75	135.81	< 0.0001
X_1_X_5_	1.54	1	1.54	0.23	0.6823
X_2_X_3_	34.34	1	34.34	5.08	0.0014
X_2_X_4_	972.00	1	972.00	143.68	< 0.0001
X_2_X_5_	147.70	1	147.70	21.83	< 0.0001
X_3_X_4_	62.11	1	62.11	9.18	< 0.0001
X_3_X_5_	422.45	1	422.45	62.45	< 0.0001
X_4_X_5_	4029.67	1	4029.67	595.66	< 0.0001
Residual	128.86	36	3.58		
Lack of fit	27.38	6	4.56	1.35	0.2669
Error	101.48	30	3.38		
Sum	37,708.24	29			

^a^Notes are the same as Table 1

The coefficients of the independent variables in the equation reflect the degree of influence of the independent variable in the equation, i.e. the degree of contribution. As seen in the regression model, the equation coefficients $K\left( {X_{2} } \right) = 133.95 > K\left( {X_{1} } \right) = 96.97 > K\left( {X_{3} } \right) = 87.31 > K\left( {X_{5} } \right) = 78.08 > K\left( {X_{4} } \right) = 31.60,$ which indicate that the degree of contribution of each ingredient to the yield is as follows: X_2_ (corn cob) > X_1_ (sawdust) > X_3_ (soybean straw) > X_5_ (corn straw) > X_4_ (rice straw). This result indicated that corncobs have the greatest degree of contribution to the yield of *G. frondosa* and can replace sawdust to serve as the main cultivation substrate of *G. frondosa*. The Pearson correlation coefficients between each of the substrates and the yield determined by the SPSS software were as follows: corn cob (0.665) > soybean straw (0.112) > sawdust (0.084) > corn straw (− 0.145) > rice straw (− 0.706). The yield was positively correlated with sawdust, corn cob and soybean straw, among which a significantly positive correlation existed between the yield and corn cob. However, yield was negatively correlated with the rice straw and corn straw, among which a significantly negative correlation existed between the yield and rice straw. The details can be seen in Table 4.

**Table 4:** Correlation analysis between yield and the different ingredients

	X_1_^a^	X_2_^a^	X_3_^a^	X_4_^a^	X_5_^a^
Yield^b^					
Pearson	0.084	0.665**	0.112	− 0.706**	− 0.145
Significance	0.767	0.008	0.692	0.003	0.607
N	21	21	21	21	21

^a^Notes are the same as Table 1

^b^Mean values followed by no letters are not significantly different at a level of 5% (P < 0.05)

** Significantly different at a level of 1% (P<0.01)

The coefficients of the mixed schemes in the equation indicate the degree of influence of the different substrate combinations on the yield. The results showed that

$$ K \, \left( {X_{2} X_{4} } \right)\, = \,110.01\, > \,K \, \left( {X_{3} X_{5} } \right)\, = \,73.71\, > \,K \, \left( {X_{2} X_{5} } \right)\, = \,44.18\, > \,K \, \left( {X_{3} X_{4} } \right)\, = \,29.74\, > \,K \, \left( {X_{1} X_{3} } \right)\, = \,16.90\, > \,K \, \left( {X_{1} X_{2} } \right)\, = \,14.76\, > \,K \, \left( {X_{1} X_{5} } \right)\, = \, - 2.17\, > \,K \, \left( {X_{2} X_{3} } \right)\, = \, - 18.16\, > \,K \, \left( {X_{1} X_{4} } \right)\, = \, - 102.94\, > \,K \, \left( {X_{4} X_{5} } \right)\, = \, - 217.52. $$

Accordingly, the degree of contribution was as follows: X_2_X_4_ (corn cob and rice straw) > X_3_X_5_ (soybean straw and corn straw) > X_2_X_5_ (corn cob and corn straw) > X_3_X_4_ (soybean straw and rice straw) > X_1_X_3_ (sawdust and soybean straw) > X_1_X_2_ (sawdust and corn cob) > X_1_X_5_ (sawdust and corn straw) > X_2_X_3_ (corn cob and soybean straw) > X_1_X_4_ (saw dust and rice straw) > X_4_X_5_ (rice straw and corn straw). Combined with the interaction analysis, mixing corn cob with sawdust, soybean straw, rice straw or corn straw significantly affected yield, and the yield increases with the corresponding increase of corn cob. Conversely, mixing rice straw with saw dust, corn cob, soybean straw or corn straw significantly reduced yield, and the yield decreases with the corresponding increase of rice straw (Table 2).

### Effects of different straws on the growth cycle of *G. frondosa*

The growth cycles for each formula (Table 5), the regression model between the growth cycle and each ingredient is as follows:

$$ Y\, = \,63.00X_{1} \, + \,69.14X_{2} \, + \,70.33X_{3} \, + \,70.00X_{4} \, + \,66.40X_{5} - 11.29X_{1} X_{2} \, + \,2.58X_{1} X_{3} \, + \,14.00X_{1} X_{4} \, + \,5.20X_{1} X_{5} - 5.62X_{2} X_{3} - 19.88X_{2} X_{4} - 1.08X_{2} X_{5} - 24.66X_{3} X_{4} - 12.94X_{3} X_{5} \, + \,127.20X_{4} X_{5} (R^{2} \, = \,0.9652) $$

**Table 5:** Measured values and predicted values of the growth cycle

Formulas^1^	Growth cycle (d)		Formulas	Growth cycle (d)	
	Measured values^2^	Predicted values		Measured values	Predicted values
1 (CK)	63.00 ± 0.0^a^	63.00	12	67.50 ± 3.10^cdefg^	67.50
2	69.14 ± 2.27^efg^	69.14	13	64.00 ± 0.00^ab^	64.00
3	70.33 ± 1.29^g^	70.33	14	65.13 ± 2.59^abc^	65.13
4	70.00 ± 0.00^fg^	70.00	15	100.00 ± 0.00^i^	100
5	66.40 ± 3.10^bcde^	66.40	16	66.65 ± 3.00^bcd^	66.65
6	63.58 ± 0.52^ab^	63.25	17	67.30 ± 3.10^cdef^	67.30
7	67.31 ± 2.59^cdef^	67.31	18	67.76 ± 3.20^cdefg^	67.76
8	70.00 ± 0.00^fg^	70.00	19	74.46 ± 3.00^gh^	74.46
9	66.00 ± 2.24^bcd^	66.00	20	73.74 ± 2.90^gh^	73.74
10	68.33 ± 4.23^defg^	68.33	21	70.71 ± 2.80^g^	70.71
11	64.60 ± 1.90^abc^	64.60			

^1^Notes are the same as Table 1

^2^Means followed by the same letter are not significantly different at a level of 5% (P < 0.05)

With the above regression equation, the predicted growth cycle can be calculated for each formula, and the measured growth cycle and the predicted growth cycle were basically the same. The statistical analysis showed that the P values of the mixed linear model and the quadratic regression model were both less than 0.0001, indicating that these two models both had a very significant level (P < 0.01) and fitted the relation between ingredients and growth cycle well. In addition, the correlation coefficient (R^2^ = 0.9652) indicated that the equation model had 96.52% goodness-of-fit with the experimental data (Tables 5 and 6).

**Table 6:** Variance analysis of the quadratic polynomial regression model for the growth cycle

Variation sources	Sum of squares	DF	Mean square	F	P
Model	3407.93	14	243.42	71.35	< 0.0001
Linear mixed model	733.34	4	183.34	53.73	< 0.0001
X_1_X_2_^a^	16.54	1	16.54	4.85	0.0342
X_1_X_3_	0.86	1	0.86	0.25	0.6178
X_1_X_4_	25.43	1	25.43	7.45	0.0097
X_1_X_5_	3.51	1	3.51	1.03	0.3173
X_2_X_3_	4.10	1	4.10	1.20	0.2805
X_2_X_4_	51.26	1	51.26	15.02	0.0004
X_2_X_5_	0.15	1	0.15	0.044	0.8344
X_3_X_4_	78.88	1	78.88	23.12	< 0.0001
X_3_X_5_	21.72	1	21.72	6.37	0.0162
X_4_X_5_	2098.92	1	2098.92	615.18	< 0.0001
Residual	122.83	36	3.41		
Lack of fit	0.00008	6	0.00001	0.000003	1.0000
Error	122.83	30	4.09		
Sum	12,865.92	44			

^a^Notes are the same as Table 1

The regression equation showed that for the equation coefficients, $K\left( {X_{3} } \right)\, = \,70.33\, > \,K\left( {X_{4} } \right)\, = \,70.00\, > \,K\left( {X_{2} } \right)\, = \,69.14\, > \,K\left( {X_{5} } \right)\, = \,66.40\, > \,K\left( {X_{1} } \right)\, = \,63.00;$ that is, the degree of contribution of each substrate to the growth cycle is X_3_ (soybean straw) > X_4_ (rice straw) > X_2_ (corn cob) > X_5_ (corn straw) > X_1_ (sawdust), indicating that the contribution of crop straw to the growth cycle is greater than that of the sawdust. Correlation in terms of how each substrate impacted the growth cycle analyzed by SPSS software, and the Pearson correlation coefficients between the substrate and the growth cycle were as follows: rice straw (0.334) > corn straw (0.235) > soybean straw (− 0.126) > corn cob (− 0.168) > sawdust (0.283). Among these, the rice straw and corn straw were both positively correlated with the growth cycle; that is, the rice straw and corn straw could extend the growth cycle of *G. frondosa*. The growth cycle was negatively correlated with the sawdust, corn cob and soybean straw, indicating that these three types of substrates can shorten the growth cycle of *G. frondosa*, with sawdust significantly shortening the growth cycle of *G. frondosa*. Details can be seen in Tables 5 and 7.

**Table 7:** Correlation analysis between the growth cycle and different ingredients

	X_1_^a^	X_2_^a^	X_3_^a^	X_4_^a^	X_5_^a^
Growth cycle^b^					
Pearson	− 0.283**	− 0.168	− 0.126	0.334**	0.235
Significance	0.213	0.468	0.586	0.139	0.305
N	21	21	21	21	21

^a^Notes are the same as Table 1

^b^Mean values followed by no letters are not significantly different at a level of 5% (P < 0.05)

** Significantly different at a level of 1% (P<0.01)

The equation coefficients of the mixed schemes showed the degree to which the different straw combinations impacted the growth cycle. The results showed that

$$ K\left( {X_{4} X_{5} } \right)\, = \,127.20\, > \,K\left( {X_{1} X_{4} } \right)\, = \,14.00\, > \,K\left( {X_{1} X_{5} } \right)\, = \,5.20\, > \,K\left( {X_{1} X_{3} } \right)\, = \,2.58\, > \,K\left( {X_{2} X_{5} } \right)\, = \, - 1.08\, > \,K\left( {X_{2} X_{3} } \right)\, = \, - 5.62\, > \,K\left( {X_{1} X_{2} } \right)\, = \, - 11.29\, > \,K\left( {X_{3} X_{5} } \right)\, = \, - 12.94\, > \,K\left( {X_{2} X_{4} } \right)\, = \, - 19.88\, > \,K\left( {X_{3} X_{4} } \right)\, = \, - 24.66. $$

The degree of contribution was X_4_X_5_ (rice straw and corn straw) > X_1_X_4_ (sawdust and rice straw) > X_1_X_5_ (sawdust and corn straw) X_1_X_3_ (sawdust and soybean straw) > X_2_X_5_ (corn cob and corn straw) > X_2_X_3_ (corn cob and soybean straw) > X_1_X_2_ (sawdust and corn cob) > X_3_X_5_ (soybean straw and corn straw) > X_2_X_4_ (corn cob and rice straw) > X_3_X_4_ (soybean straw and rice straw). When combined with the interaction analysis, the results showed that mixing rice straw with the sawdust, corn cob, soybean straw or corn straw could significantly increase the growth cycle, and the life cycle increased with the increase of rice straw (Table 5).

### High-yielding formula and verification test

**Fig. 1:**
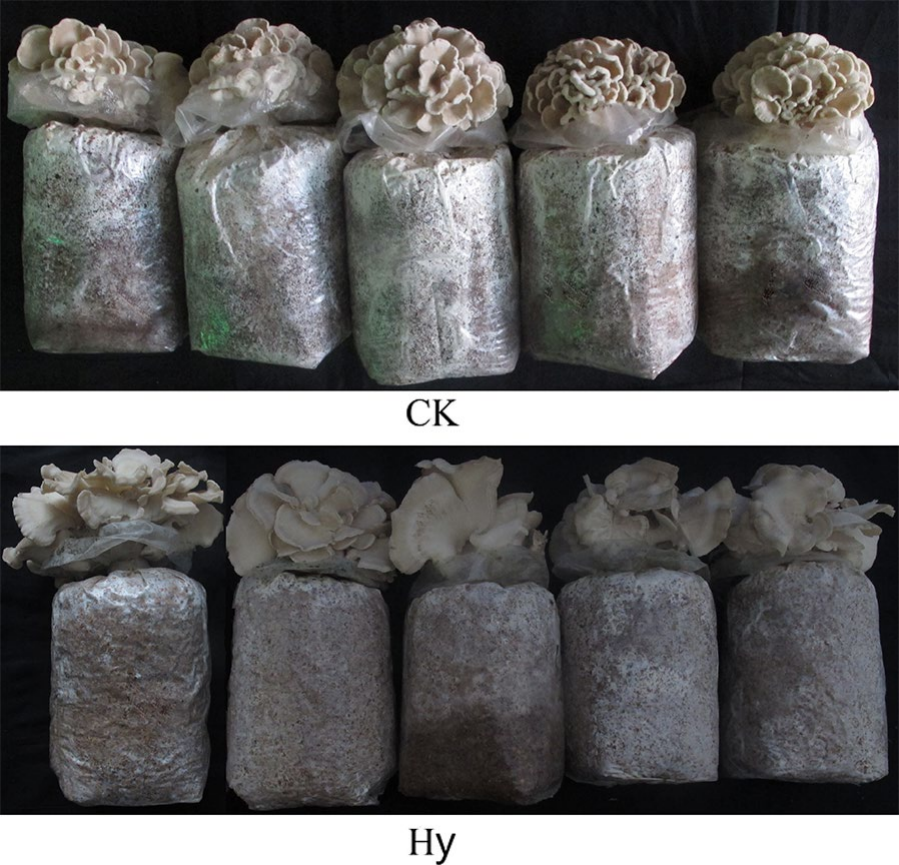
*Grifola frondosa* fruiting in the high-yield formula (Hy) and the control (CK)

Using the yield as the evaluation index and setting the variation range and expected response value of each ingredient, steepest slope prediction was carried out based on the regression equation beginning from a random combination, and a high-yielding formula with no sawdust was generated. The formula was as follows: corn cob (X_2_) 97.5% and rice straw (X_4_) 2.5%, which were multiplied by the coefficient of 0.75 and converted to 73.125% corn cob and 1.875% rice straw, respectively, combined with 23% wheat bran and 2% light CaCO_3_ (C/N = 48.40). The predicted yield was 134.07 g/bag. The measured yield of the first flush reached 134.72 ± 4.24 g/bag, which basically verified the predicted fitted value. The results showed that the formula had a yield 39.97% higher than the control, with a BE of 44.91 ± 1.41%. In addition to yield and BE, single-fruiting-body length and width, the growth cycle of the high-yielding formula was also examined. The single-fruiting-body length and width of *G. frondosa* for the high-yield (5.15 and 3.45 respectively) formula were higher than those for the control (2.58 and 2.34 respectively), but the growth cycle was longer than that of the control (Table 8 and Fig. 1).

**Table 8:** Comparison of the main agronomic traits between the high-yield straw-substrate formula and the control

Formulas	Single-fruiting-body length (cm)^2^	Single-fruiting-body width (cm)	Yield (g)		Growth cycle (d)		BE (%)
			Predicted value	Measured value	Predicted value	Measured value	
1 (CK)	2.58 ± 0.37^b^	2.34 ± 0.37^b^	96.97	97.25 ± 9.71^b^	63.00	63.00 ± 0.0^a^	32.42 ± 3.24^b^
Hy^1^	5.15 ± 0.148^a^	3.45 ± 0.24^a^	134.07	134.72 ± 4.24^a^	68.68	68.68 ± 2.32^b^	44.91 ± 1.41^a^

^1^Hy high-yield

^2^Mean values followed by no letters are not significantly different at a level of 5% (P < 0.05)

## Discussion

Interest in *G. frondosa* production has increased significantly in the last decade due to their nutritional and health-promoting benefits (Vetvicka and Vetvickova 2014; Ding et al. 2016). *G. frondosa* is traditionally cultivated on forest wood (sawdust) however due to recent efforts in managing forest resources the use of agro-forestry products for its cultivation is not sustainable. Most edible fungi can be cultivated with agro-residues such as crop straws (Barreto et al. 2008; Philippoussis 2009) either by composting or non-composting. However, due to the differences in nutritional requirements, growth specificity, inheritance characteristics and activities of the secreted extracellular enzymes among edible fungi (Montoya et al. 2012; Yang et al. 2013a), differences exist in the utilization capacity of the different crop straws. In this work, we investigated the use of different crop straw (corn cob, soybean straw, rice straw and corn stalk) as alternate substrate to sawdust for cultivating *G. frondosa* and developed a statistical model for optimizing production and evaluated its effect on yield and growth cycle.

In our work, all substrate formulae contained 23% wheat bran and 2% light CaCO_3_. Substrates supplemented with wheat bran produces better quality mushrooms (Moonmoon et al. 2011) while the limited amount of CaCO_3_ improves calcium metabolism, adjust pH of substrates and makes mushroom production more consistent from crop to crop (Royse and Sanchez-Vazquez 2003).

This study showed that in optimizing the formula of *G. frondosa* production, the yield could be increased by adding a certain proportion of corn cob (50%), such as used in formula 6, which had a yield of 22.37% higher than the control. The use of rice straw negatively correlated with yield; however, it can still be increased by the addition of a certain proportion (50%) of corn cob to rice straw, such as used in formula 11. For instance, formula 11 (110.00 ± 11.41 g) showed a yield increase of 246.45% compared with formula 4 (31.75 ± 3.61 g), which had rice straw alone, and showed a yield increase of 13.72% compared with the control group (Table 2). Therefore, in places where the rice resources are rich, adding an appropriate proportion of corn cob to replace sawdust can achieve the same effect in *G. frondosa* cultivation. Soybean straw and corn straw in a reasonable mixture ratio, or combined with substrates such as corn cob, can also replace sawdust to achieve the same effect in cultivation. When the yield is considered the priority indicator in the production of *G. frondosa*, using corn cob powder as the main cultivation substrate substitute for sawdust will not only increase the yield but also increase the single-fruiting-body area.

When the growth cycle is used as the sole evaluation index, sawdust, soybean straw and corn cob can significantly shorten the growth cycle of *G. frondosa*, with sawdust having the greatest effect. For instance, with formula 6, which had 37.5% sawdust and 37.5% corn cob, the growth cycle was nearly the same as that in control, and its yield was higher than that in control. Although *G. frondosa* had a long growth cycle with the soybean straw substrate, the growth cycle can be significantly shortened by combining soybean straw with rice straw and corn straw, as evidenced in formulae 13 and 14 (Table 5). Therefore, when taking both growth cycle and yield into account, a certain percentage of sawdust can be added to the straw formula to shorten the growth cycle and increase the yield of *G. frondosa* as well. Since lignin content in sawdust was higher than that in crop straws, the substrate with high lignin content is observed to promote mycelial growth and thus shorten the growth cycle. The rapid colonization of substrate during spawn run prevents competition from other competitive molds and pathogenic microorganisms. Also, the shortened duration of the growth cycle saves costs, improve income and it is suitable for commercial producers of *G. frondosa* (Gaitan-Hernandez et al. 2006).

The effects of different ingredients on the agronomic traits of *G. frondosa* were different. This can be attributed to their nutrient composition and structure of the different ingredient. The materials used in the cultivation medium, such as sawdust, corn cob and cottonseed shell are composed of lignin, cellulose and minerals, which provides sufficient carbon and nitrogen sources for the growth and development of edible fungi (Naraian et al. 2009). In accordance with the production needs, these ingredients can be mixed to achieve the desired goal of cultivation. The C/N ratio as an index must be considered in the choice of raw materials to include substrate formulation because it plays a vital role in the growth of edible fungi.

The study showed that substrates with low carbon–nitrogen ratios result in significant reduction in the yield of *Grifola frondosa,* observed with formulae 3–5, 13, 15, and 18–21. Also higher carbon and nitrogen ratios did not improve *G. frondosa* yield, as with the use of sawdust in formula 1. However, some of the low carbon–nitrogen formulations yield higher than control, such as formula 10 (C/N = 28.65) and the formula 14 (C/N = 25.22). A high carbon–nitrogen ratio is beneficial to the growth of mycelia, and thus shortens the growth period of *G. frondosa*, as in formulae 1 (C/N = 74.64) and 6 (C/N = 59.81), the shortest growth period, of 63 and 63.58 days respectively (Tables 1, 5). Low carbon and nitrogen sources leads to premature senescence and slow mycelial growth respectively. On the other hand, high nitrogen levels lead to vigorous mycelial growths resulting in the accumulation of unwanted metabolic products, which affected the yield (Naraian et al. 2009; Hoa et al. 2015). These findings were consistent to the results reported by Naraian (Naraian et al. 2009). Therefore a reasonable C/N ratio plays a crucial role in the yield and growth cycle of edible fungi (Naraian et al. 2009; Hoa et al. 2015).

The results of this study can be directly applied in the industrial production of *G. frondosa*, which is more suitable for the developmental requirements of the edible fungi industry. However, formulae and techniques need to be optimized, and the economic balance needs to be improved before commercial development is achieved (Gaitan-Hernandez et al. 2006). Hence, the need to use a statistical method to optimize and standardize substrate mixtures for optimal growth and yield.

The use of the simplex-lattice method for formula design has been less applied in the field of edible fungi (Yang et al. 2016). However, as the most basic design scheme in the mixture design, this method is widely used in feed, medicine and food industries because the test points can be taken on the lattice points in the regular simplex to ensure that the test points are evenly distributed and that the calculation is simple and accurate since the regression coefficient is only a simple function of the response values at the corresponding lattice points. In this study, by using the simplex-lattice design, the purpose of testing the effects of different mixture ratios of crop straws and sawdust on the cultivation of *G. frondosa* was achieved; this was affirmed by the high R^2^ value (0.956), indicating the percentage of the variability explained by the model (Table 1). In all cases the R^2^ was higher than 0.8, with values over ~ 0.9 in almost all responses.

Consequently, the optimized formula obtained was effective, and the yield and growth cycle equations allow effective prediction of the production effect of the formula. Therefore, the simplex-lattice design can be applied designing experiments for the formulation of substrates for edible fungi cultivation.

Our results show that corn cob was the best alternative to sawdust as a substrate for producing a high yield of *G. frondosa*. This is because corn cob does not only provide nutrients for the growth but as a substrate, it supports and facilitates the attachment of the fungi and allows oxygen transfer, heat dispersion and has the suitable C/N ratio to promote the induction of fruiting bodies among other conditions (Hoa et al. 2015; Pérez-Rodríguez et al. 2016). Compared to rice straw, corn cob has high lignin content, certain proportions of soluble polysaccharides, which can be directly utilized by the mycelia, and it is beneficial to the growth of edible fungi.

Corn cob was considered the best agricultural residue among all the other common studied wastes (Naraian et al. 2009). Because corn is widely grown in the world, the corn cob is abundant, readily available and cheap, a notable percentage makes up the by-product of maize processing (Niu et al. 2016) and has been widely used in industry and agriculture. Its composition indicates the potential utilization as substrate for fungal growth as well as the production of industrial enzymes such as xylanase and xylose among others (Amaro-Reyes et al. 2016; Kashid and Ghosalkar 2017).

In conclusion, corn cob significantly increases the yield of *G. frondosa* and can replace sawdust as the main cultivation substrate of *G. frondosa*, but sawdust significantly shortens the growth cycle of *G. frondosa*. The optimized high-yield formula was 73.125% corn cob, 1.875% rice straw, 23% wheat bran, and 2% light CaCO_3_ (C/N = 48.40); the average yield of the first flush was 134.72 ± 4.24 g/bag, and the BE was 44.91 ± 1.41%. The results of this study reinforce the use of crop straw for cultivation of *G. frondosa* and provide a scientific basis for the production of *G. frondosa* using corn cob as the main substrate.

## Abbreviations

ANOVA: one-way analysis of variance
BE: biological efficiency
C/N: carbon to nitrogen ratio
SPSS: statistical product and service solutions
